# Supplementary material for: Telomere Length as a New Risk Marker of Early-Onset Colorectal Cancer
Source: Int J Mol Sci. 2023 Feb 9;24(4):3526. doi: 10.3390/ijms24043526 (PMC9965248; doi:10.3390/ijms24043526)
Supplement: Supplementary file 1 [file ijms-24-03526-s001.zip › ijms-2115892-supplementary.pdf]

SUPPLEMENTARY INFORMATION

Telomere Length as a New Risk Marker of Early-Onset Colorectal Cancer

Abel Martel-Martel, Luis A. Corchete, Marc Martí, Rosario Vidal-Tocino, Elena Hurtado, Edurne Álvaro, Fernando Jiménez, Marta Jiménez-Toscano, Francesc Balaguer, Gonzalo Sanz, Irene López, Sergio Hernández-Villafranca, Araceli Ballestero, Alfredo Vivas, Sirio Melone, Carlos Pastor, Lorena Brandáriz, Manuel A. Gómez-Marcos, Juan J. Cruz-Hernández, José Perea * and Rogelio González-Sarmiento *

Supplementary Table S1. Early-Onset colorectal cancer cases clinical data and their impact on telomere length.

Characteristic	Total (%)	TL Mean (kb)	*p*-Value
**Total**	87		
**Sex**			
Female	36 (41)	136	0.140
Male	51 (59)	114	
**Age at Diagnosis**			
<29 years	4 (5)	220.6	0.096
(30-39) years	15 (17)	98	
(40-50) years	68 (78)	122.1	
Mean (std), years	42.7 (6.2)		
Median (range), years	44 (23-49)		
**Body Mass Index (BMI)**			
Underweight (<18.5 kg/m2)	2 (2)	126	0.268
Normoweight (18.5-<25 kg/m2)	33 (38)	149	
Overweight (25-<30 kg/m2)	24 (28)	106	
Obese (>30 kg/m2)	11 (13)	113	
Unknown	17 (19)		
Mean (std), kg/m2	25.15 (5.4)		
Median (range), kg/m2 3	24.9 (15.6-43)		
**Tumor Stage at diagnosis**			
I	13 (15)	104	0.803
II	18 (21)	122	
III	32 (37)	122	
IV	22 (23)	129	
**Tumor Site**			
Right colon	20 (23)	112	0.472
Left colon	27 (31)	134	
Rectosigmoid junction/Rectum	40 (46)	119	
**Histological features***			
Mucinous	13/59 (22)	118/124	0.812
"Signet ring" cells	4/59 (7)	113/123	0.820
Unknown	18 (21)		
**Grade of differentiation***			
High	9 (10)	89	0.067
Medium	23 (26)	117	
Low	27 (31)	138.3	
Unknown	18 (21)		

**Multiple Primary Neoplasms**			
Synchronous colorectal cancer	3 (3)	61.8	0.065
Other Multiple Primary Neoplasms	8 (9)	125	0.584
**Familial History of Colorectal Cancer**			
Yes	19 (22)	145	0.345
No	60 (69)	120	
Unknown	8 (9)		
**Sporadic cases**			
Yes	39 (45)	113	0.126
No	43 (49)	136	
Unknown	5 (6)		

* The remaining cases are those with only biopsy due to palliative conditions.
